# Supplementary material for: Epidemiological Impact of Increasing Vaccination Coverage Rate and Re-Vaccination on Pneumococcal Disease in Older Adults in Germany
Source: Vaccines (Basel). 2025 Apr 28;13(5):475. doi: 10.3390/vaccines13050475 (PMC12115568; doi:10.3390/vaccines13050475)
Supplement: Supplementary file 1 [file vaccines-13-00475-s001.zip › vaccines-3562253-SI.pdf]

Supplementary Materials

TABLE OF CONTENTS

Model Equations 2

Dynamic Transmission Model 2

Model Calibration 13

Calibrated Parameters 13

Model Fits 15

Vaccine Efficacy 16

IPD 16

NBPP 17

Additional Model Results 19

IPD 19

Inpatient NBPP 21

Outpatient NBPP 22

References 24

Model Equations

Demographic parameters are chosen according to Hethcote et al. [1].

Dynamic Transmission Model

Figure S1. Flow diagram for dynamic transmission model.

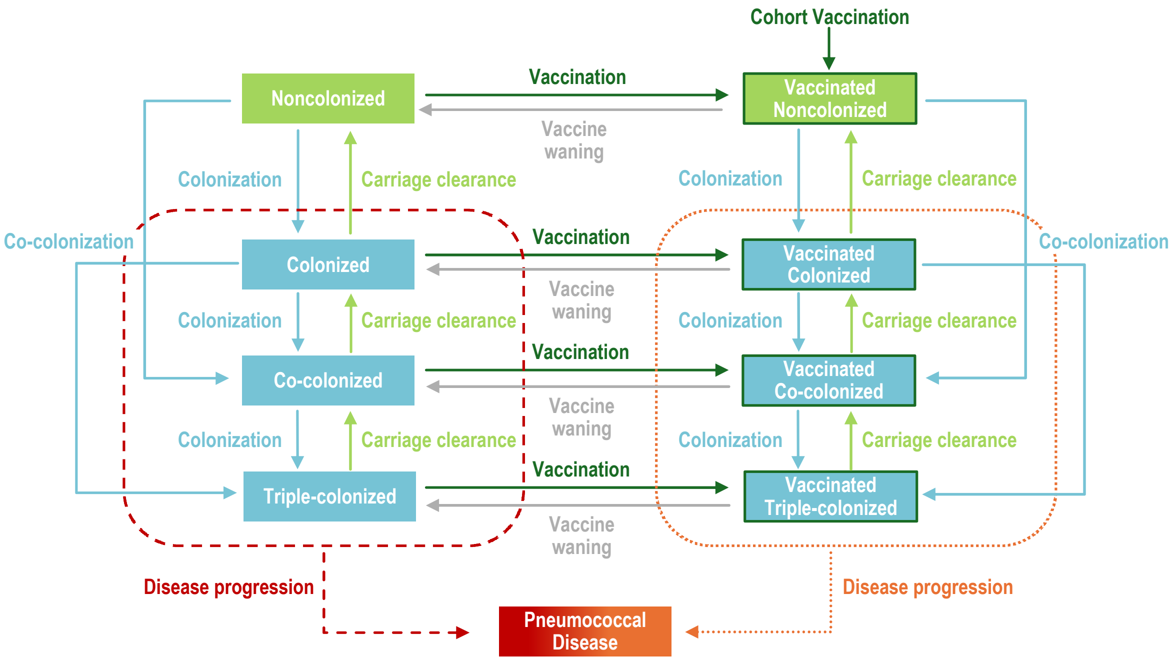

The definitions for the epidemiological parameters and their values used for our analysis are provided in Tables S1 and S2.

Table S1. Description of model compartments and population.

**Variable**	**Description**
$\mathcal{N}_{u,a}$	Non-colonized unvaccinated individuals in age group a.
$\mathcal{N}_{\sigma,a}$	Non-colonized and σ-vaccinated individuals in age group a.
$\mathcal{C}_{u,a,i}$	Unvaccinated individuals in age group a colonized with STC i.
$\mathcal{C}_{\sigma,a,i}$	σ-vaccinated individuals in age group a colonized with STC i.
$\mathcal{CC}_{u,a,i,j}$	Unvaccinated individuals in age group a co-colonized with STCs i and j.
$\mathcal{CC}_{\sigma,a,i,j}$	σ-vaccinated individuals in age group a co-colonized with STCs i and j.
$\mathcal{CCC}_{u,a,i,j,k}$	Unvaccinated individuals in age group a co-colonized with STCs i, j, and k.
$\mathcal{CCC}_{\sigma,a,i,j,k}$	σ-vaccinated individuals in age group a co-colonized with STCs i, j, and k.
$\mathcal{Z}_{\sigma,a}$	Previously vaccinated individuals who have waned σ-vaccine in age group a and are non-colonized.
$\mathcal{Z}_{\sigma,a,i}$	Previously vaccinated individuals who have waned σ-vaccine in age group a colonized with STC i.

$\mathcal{ZZ}_{\sigma,a,i,j}$	Previously vaccinated individuals who have waned σ-vaccine in age group a colonized with STCs i and j.
$\mathcal{ZZZ}_{\sigma,a,i,j,k}$	Previously vaccinated individuals who have waned σ-vaccine in age group a colonized with STCs i, j, *and* k.
$\mathcal{P}_a$	Total population in age group a.

Table S2. Description of epidemiological model parameters.

Parameter	Description	Source
$c_{a,b}$	Average number of contacts an individual in age group a makes with an individual in age group b per year.	Data
$\beta_{a,i}$	Probability of acquisition of carriage of STC i per contact in age group a.	Calibrated – Transmission model
$\gamma_{a,i}$	STC i clearance rate of individuals in age group a. Note that this parameter can be parameterized to vary according to vaccine history.	Data
$\theta_{i,j}$	Competition parameter if currently colonized with STC i (risk reduction for second colonization with j).	Calibrated – Transmission model
$\rho_{a,i}$	Probability of developing a pneumococcal disease given carriage with STC i in age group a, i.e., the case-to-carrier ratio.	Calibrated – Transmission model
$\epsilon_{\sigma,a,i}$	σ-vaccine efficacy against carriage acquisition of STC i in age group a.	Calibrated – Transmission model
$\epsilon_{D,\sigma,a,i}$	σ-vaccine efficacy against developing pneumococcal disease from STC i in age group a.	Data
ω_σ	σ-vaccine waning rate. Note that this parameter can be parameterized to vary by age.	Data
$\alpha_{a,b}$	Transmissibility of carriage from age group b to age group a.	Defined
σ	Vaccination with either PPSV (w) or PCV (v) or sequential PCV and PPSV (vw).	Defined
$\phi_{v,a}, \phi_{w,a}$	Cohort rate of PCV or PPSV vaccination of the unvaccinated individuals in age group a.	Calibrated – Vaccine model
$\psi_{v,a}, \psi_{w,a}$	Continuous rate of PCV or PPSV vaccination of the unvaccinated individuals in age group a.	Calibrated – Vaccine model
$\psi v_{w,a}, \psi w_{v,a}$	Continuous rate of PCV or PPSV vaccination of those who were previously vaccinated with the other vaccine (PPSV or PCV, respectively).	Calibrated – Vaccine model
$\zeta_{v,a}$, $\zeta_{w,a}$	Continuous rate of PCV or PPSV vaccination of a sequentially (PCV and PPSV) waned individual in age group a.	Calibrated – Vaccine model
$\xi_{v,a}, \xi_{w,a}$	Continuous rate of PCV or PPSV vaccination of a PPSV waned individual in age group a.	Calibrated – Vaccine model
$\chi_{v,a}, \chi_{w,a}$	Continuous rate of PCV or PPSV vaccination of a PCV waned individual in age group a.	Calibrated – Vaccine model

$\zeta c_{v,a}, \zeta c_{w,a}$	Cohort rate of PCV or PPSV vaccination of a sequentially (PCV and PPSV) waned individual in age group a.	Calibrated – Vaccine model
$\xi c_{v,a}, \xi c_{w,a}$	Cohort rate of PCV or PPSV vaccination of a PPSV waned individual in age group a.	Calibrated – Vaccine model
$\chi c_{v,a}, \chi c_{w,a}$	Cohort rate of PCV or PPSV vaccination of a PCV waned individual in age group a.	Calibrated – Vaccine model

The nonlinear differential equations that govern the flows in our model are specified as follows:

$$\mathcal{N}'_{u,a} = \delta_{1,a}\Lambda(1-\phi) + (1-\delta_{1,a})(1-\phi_{v,a}-\phi_{w,a})m_{a-1}\mathcal{N}_{u,a-1} + \sum_{i=1}^{nS}\gamma_{u,a,i}\mathcal{C}_{u,a,i}$$

$$-\left(\mu_a + m_a + \psi_{v,a} + \psi_{w,a} + \sum_{i=1}^{nS}\lambda_{a,i} + \sum_{i=1}^{nS-1}\sum_{j=i+1}^{nS}\lambda_{a,i,j}\right)\mathcal{N}_{u,a},$$

$$\mathcal{N}'_{v,a} = \delta_{1,a}\Lambda\phi + (1-\delta_{1,a})(1-\phi v_{w,a})m_{a-1}\mathcal{N}_{v,a-1} + (1-\delta_{1,a})\phi_{v,a}m_{a-1}\mathcal{N}_{u,a-1}$$

$$+ (1-\delta_{1,a})\chi c_{v,a}m_{a-1}\mathcal{Z}_{v,a-1} + (1-\delta_{1,a})\xi c_{v,a}m_{a-1}\mathcal{Z}_{w,a-1}$$

$$+ (1-\delta_{1,a})\zeta c_{v,a}m_{a-1}\mathcal{Z}_{vw,a-1} + \psi_{v,a}\mathcal{N}_{u,a} + \chi_{v,a}\mathcal{Z}_{v,a} + \xi_{v,a}\mathcal{Z}_{w,a} + \zeta_{v,a}\mathcal{Z}_{vw,a}$$

$$+ \sum_{i=1}^{nS}\gamma_{v,a,i}\mathcal{C}_{v,a,i} - \left(\mu_a + m_a + \omega_{v,a} + \psi v_{w,a} + \sum_{i=1}^{nS}\lambda v_{v,a,i} + \sum_{i=1}^{nS-1}\sum_{j=i+1}^{nS}\lambda v_{v,a,i,j}\right)\mathcal{N}_{v,a},$$

$$\mathcal{N}'_{w,a} = (1-\delta_{1,a})m_{a-1}(1-\phi w_{v,a})\mathcal{N}_{w,a-1} + (1-\delta_{1,a})\phi_{w,a}m_{a-1}\mathcal{N}_{u,a-1}$$

$$+ (1-\delta_{1,a})\chi c_{w,a}m_{a-1}\mathcal{Z}_{v,a-1} + (1-\delta_{1,a})\xi c_{w,a}m_{a-1}\mathcal{Z}_{w,a-1}$$

$$+ (1-\delta_{1,a})\zeta c_{w,a}m_{a-1}\mathcal{Z}_{vw,a-1} + \psi_{w,a}\mathcal{N}_{u,a} + \chi_{w,a}\mathcal{Z}_{v,a} + \xi_{w,a}\mathcal{Z}_{w,a} + \zeta_{w,a}\mathcal{Z}_{vw,a}$$

$$+ \sum_{i=1}^{nS}\gamma_{w,a,i}\mathcal{C}_{w,a,i} - \left(\mu_a + m_a + \omega_{w,a} + \psi w_{v,a} + \sum_{i=1}^{nS}\lambda v_{w,a,i}\right.$$

$$\left. + \sum_{i=1}^{nS-1}\sum_{j=i+1}^{nS}\lambda v_{w,a,i,j}\right)\mathcal{N}_{w,a},$$

$$\mathcal{N}'_{vw,a} = (1-\delta_{1,a})m_{a-1}\mathcal{N}_{vw,a-1} + (1-\delta_{1,a})\phi v_{w,a}m_{a-1}\mathcal{N}_{v,a-1} + (1-\delta_{1,a})\phi w_{v,a}m_{a-1}\mathcal{N}_{w,a-1} + \psi v_{w,a}\mathcal{N}_{v,a} + \phi w_{v,a}\mathcal{N}_{w,a} + \sum_{i=1}^{nS}\gamma_{vw,a,i}\mathcal{C}_{vw,a,i} - \left(\mu_a + m_a + \omega_{vw,a} + \sum_{i=1}^{nS}\lambda v_{vw,a,i} + \sum_{i=1}^{nS-1}\sum_{j=i+1}^{nS}\lambda v_{vw,a,i,j}\right)\mathcal{N}_{vw,a}.$$

The age-specific population that was singly colonized by STC i was either unvaccinated ($\mathcal{C}_{u,a,i}$), vaccinated with a PCV ($\mathcal{C}_{v,a,i}$), vaccinated with a PPSV ($\mathcal{C}_{w,a,i}$), or vaccinated sequentially with both a PCV and PPSV ($\mathcal{C}_{vw,a,i}$) is represented by the following set of ODEs,

$$\mathcal{C}'_{u,a,i} = (1-\delta_{1,a})(1-\phi_{v,a}-\phi_{w,a})m_{a-1}\mathcal{C}_{u,a-1,i} + \lambda_{a,i}\mathcal{N}_{u,a} + \sum_{j=i+1}^{\mathrm{nS}}\gamma_{u,a,j}\mathcal{CC}_{u,a,i,j} + \sum_{j=1}^{\mathrm{i-1}}\gamma_{u,a,j}\mathcal{CC}_{u,a,j,i} - \left(\mu_a + m_a + \psi_{v,a} + \psi_{w,a} + \gamma_{u,a,i} + \sum_{\substack{j=1,\\ j\neq i}}^{\mathrm{nS}}\theta_{i,j}\lambda_{a,j} + \sum_{j=i+1}^{\mathrm{nS}}\theta_{i,j}\lambda_{a,i,j} + \sum_{j=1}^{\mathrm{i-1}}\theta_{i,j}\lambda_{a,j,i} + \sum_{\substack{j=1\\ j\neq i}}^{nS-1}\sum_{\substack{k=j+1\\ k\neq i}}^{nS}\theta\theta_{i,j,k}\lambda_{a,j,k}\right)\mathcal{C}_{u,a,i}$$

$$\mathcal{C}'_{v,a,i} = (1-\delta_{1,a})(1-\phi v_{w,a})m_{a-1}\mathcal{C}_{v,a-1,i} + (1-\delta_{1,a})\phi_{v,a}m_{a-1}\mathcal{C}_{u,a-1,i} + (1-\delta_{1,a})\chi c_{v,a}m_{a-1}\mathcal{Z}_{v,a-1,i} + (1-\delta_{1,a})\xi c_{v,a}m_{a-1}\mathcal{Z}_{w,a-1,i} + (1-\delta_{1,a})\zeta c_{v,a}m_{a-1}\mathcal{Z}_{vw,a-1,i} + \psi_{v,a}\mathcal{C}_{u,a,i} + \chi_{v,a}\mathcal{Z}_{v,a,i} + \xi_{v,a}\mathcal{Z}_{w,a,i} + \zeta_{v,a}\mathcal{Z}_{vw,a,i} + \lambda v_{v,a,i}\mathcal{N}_{v,a} + \sum_{j=i+1}^{\mathrm{nS}}\gamma_{v,a,j}\mathcal{CC}_{v,a,i,j} + \sum_{j=1}^{\mathrm{i-1}}\gamma_{v,a,j}\mathcal{CC}_{v,a,j,i} - \left(\mu_a + m_a + \omega_{v,a} + \psi v_{w,a} + \gamma_{v,a,i} + \sum_{\substack{j=1,\\ j\neq i}}^{\mathrm{nS}}\theta_{i,j}\lambda v_{v,a,j} + \sum_{j=i+1}^{\mathrm{nS}}\theta_{i,j}\lambda v_{v,a,i,j} + \sum_{j=1}^{\mathrm{i-1}}\theta_{i,j}\lambda v_{v,a,j,i} + \sum_{\substack{j=1\\ j\neq i}}^{nS-1}\sum_{\substack{k=j+1\\ k\neq i}}^{nS}\theta\theta_{i,j,k}\lambda \mathrm{v}_{v,a,j,k}\right)\mathcal{C}_{v,a,i}$$

$$
\begin{aligned}
\mathcal{C}'_{w,a,i} = {} & (1-\delta_{1,a})(1-\phi w_{v,a})m_{a-1}\mathcal{C}_{w,a-1,i} + (1-\delta_{1,a})\phi_{w,a}m_{a-1}\mathcal{C}_{u,a-1,i} \\
& + (1-\delta_{1,a})\chi c_{w,a}m_{a-1}\mathcal{Z}_{v,a-1,i} + (1-\delta_{1,a})\xi c_{w,a}m_{a-1}\mathcal{Z}_{w,a-1,i} \\
& + (1-\delta_{1,a})\zeta c_{w,a}m_{a-1}\mathcal{Z}_{vw,a-1,i} + \psi_{w,a}\mathcal{C}_{u,a,i} + \chi_{w,a}\mathcal{Z}_{v,a,i} + \xi_{w,a}\mathcal{Z}_{w,a,i} + \zeta_{w,a}\mathcal{Z}_{vw,a,i} \\
& + \lambda v_{w,a,i}\mathcal{N}_{w,a} + \sum_{j=i+1}^{\mathrm{nS}} \gamma_{w,a,j}\mathcal{CC}_{w,a,i,j} + \sum_{j=1}^{\mathrm{i-1}} \gamma_{w,a,j}\mathcal{CC}_{w,a,j,i} \\
& - \left(\mu_a + m_a + \omega_{w,a} + \psi w_{v,a} + \gamma_{w,a,i} + \sum_{\substack{j=1,\\ j\neq i}}^{\mathrm{nS}} \theta_{i,j}\lambda v_{w,a,j} + \sum_{j=i+1}^{\mathrm{nS}} \theta_{i,j}\lambda v_{w,a,i,j} \right. \\
& \left. + \sum_{j=1}^{\mathrm{i-1}} \theta_{i,j}\lambda v_{w,a,j,i} + \sum_{\substack{j=1\\ j\neq i}}^{nS-1} \sum_{\substack{k=j+1\\ k\neq i}}^{nS} \theta\theta_{i,j,k}\lambda \mathrm{v}_{w,a,j,k} \right) \mathcal{C}_{w,a,i}
\end{aligned}
$$

$$
\begin{aligned}
\mathcal{C}'_{vw,a,i} = {} & (1-\delta_{1,a})m_{a-1}\mathcal{C}_{vw,a-1,i} + (1-\delta_{1,a})\phi v_{w,a}m_{a-1}\mathcal{C}_{v,a-1,i} + (1-\delta_{1,a})\phi w_{v,a}m_{a-1}\mathcal{C}_{w,a-1,i} \\
& + \psi v_{w,a}\mathcal{C}_{v,a,i} + \psi w_{v,a}\mathcal{C}_{w,a,i} + \lambda v_{vw,a,i}\mathcal{N}_{vw,a} + \sum_{j=i+1}^{\mathrm{nS}} \gamma_{vw,a,j}\mathcal{CC}_{vw,a,i,j} \\
& + \sum_{j=1}^{\mathrm{i-1}} \gamma_{vw,a,j}\mathcal{CC}_{vw,a,j,i} \\
& - \left(\mu_a + m_a + \omega_{vw,a} + \gamma_{vw,a,i} + \sum_{\substack{j=1,\\ j\neq i}}^{\mathrm{nS}} \theta_{i,j}\lambda v_{vw,a,j} + \sum_{j=i+1}^{\mathrm{nS}} \theta_{i,j}\lambda v_{vw,a,i,j} \right. \\
& \left. + \sum_{j=1}^{\mathrm{i-1}} \theta_{i,j}\lambda v_{vw,a,j,i} + \sum_{\substack{j=1\\ j\neq i}}^{nS-1} \sum_{\substack{k=j+1\\ k\neq i}}^{nS} \theta\theta_{i,j,k}\lambda \mathrm{v}_{vw,a,j,k} \right) \mathcal{C}_{vw,a,i}.
\end{aligned}
$$

The age-specific population that was co-colonized by STC i and j was either unvaccinated ($\mathcal{CC}_{u,a,i}$), vaccinated with a PCV ($\mathcal{CC}_{v,a,i}$), vaccinated with a PPSV ($\mathcal{CC}_{w,a,i}$), or vaccinated sequentially with both a PCV and PPSV ($\mathcal{CC}_{vw,a,i}$) is represented by the following set of ODEs,

$$
\begin{aligned}
\mathcal{CC}'_{u,a,i,j} = {} & (1-\delta_{1,a})(1-\phi_{v,a}-\phi_{w,a})m_{a-1}\mathcal{CC}_{u,a-1,i,j} + \theta_{i,j}(\lambda_{a,j}+\lambda_{a,i,j})\mathcal{C}_{u,a,i} \\
& + \theta_{j,i}(\lambda_{a,i}+\lambda_{a,i,j})\mathcal{C}_{u,a,j} + \lambda_{a,i,j}\mathcal{N}_{u,a} + \sum_{\substack{k=1\\ i<j\\ j<k}}^{nS}\gamma_{u,a,k}\mathcal{CCC}_{u,a,i,j,k} + \sum_{\substack{k=1\\ i<k\\ k<j}}^{nS}\gamma_{u,a,k}\mathcal{CCC}_{u,a,i,k,j} \\
& + \sum_{\substack{k=1\\ k<i\\ i<j}}^{nS}\gamma_{u,a,k}\mathcal{CCC}_{u,a,k,i,j} \\
& - \left(\mu_a + m_a + \gamma_{u,a,i} + \gamma_{u,a,j} + \psi_{v,a} + \psi_{w,a} + \sum_{\substack{k=1\\ k\neq i\\ k\neq j}}^{\mathrm{nS}}\theta_{i,j,k}\lambda_{a,k} + \sum_{\substack{k=1\\ i<k\\ j\neq k}}^{\mathrm{nS}}\theta_{i,j,k}\lambda_{a,i,k}\right. \\
& \left. + \sum_{\substack{k=1\\ k<i\\ j\neq k}}^{\mathrm{nS}}\theta_{i,j,k}\lambda_{a,k,i} + \sum_{\substack{k=1\\ j<k\\ i\neq k}}^{\mathrm{nS}}\theta_{i,j,k}\lambda_{a,j,k} + \sum_{\substack{k=1\\ k<j\\ i\neq k}}^{\mathrm{nS}}\theta_{i,j,k}\lambda_{a,k,j}\right)\mathcal{CC}_{u,a,i,j}
\end{aligned}
$$

$$
\begin{aligned}
\mathcal{CC}'_{v,a,i,j} = {} & (1-\delta_{1,a})(1-\phi v_{w,a})m_{a-1}\mathcal{CC}_{v,a-1,i,j} + (1-\delta_{1,a})\phi_{v,a}m_{a-1}\mathcal{CC}_{u,a-1,i,j} \\
& + (1-\delta_{1,a})\chi c_{v,a}m_{a-1}\mathcal{ZZ}_{v,a-1,i,j} + (1-\delta_{1,a})\xi c_{v,a}m_{a-1}\mathcal{ZZ}_{w,a-1,i,j} \\
& + (1-\delta_{1,a})\zeta c_{v,a}m_{a-1}\mathcal{ZZ}_{vw,a-1,i,j} + \psi_{v,a}\mathcal{CC}_{u,a,i,j} + \chi_{v,a}\mathcal{ZZ}_{v,a,i,j} + \xi_{v,a}\mathcal{ZZ}_{w,a,i,j} \\
& + \zeta_{v,a}\mathcal{ZZ}_{vw,a,i,j} + \theta_{i,j}(\lambda v_{v,a,j}+\lambda v_{v,a,i,j})\mathcal{C}_{v,a,i} + \theta_{j,i}(\lambda v_{v,a,i}+\lambda v_{v,a,i,j})\mathcal{C}_{v,a,j} \\
& + \lambda v_{v,a,i,j}\mathcal{N}_{v,a} + \sum_{\substack{k=1\\ i<j\\ j<k}}^{nS}\gamma_{v,a,k}\mathcal{CCC}_{v,a,i,j,k} + \sum_{\substack{k=1\\ i<k\\ k<j}}^{nS}\gamma_{v,a,k}\mathcal{CCC}_{v,a,i,k,j} + \sum_{\substack{k=1\\ k<i\\ i<j}}^{nS}\gamma_{v,a,k}\mathcal{CCC}_{v,a,k,i,j} \\
& - \left(\mu_a + m_a + \omega_{v,a} + \gamma_{v,a,i} + \gamma_{v,a,j} + \psi v_{w,a} + \sum_{\substack{k=1\\ k\neq i\\ k\neq j}}^{\mathrm{nS}}\theta_{i,j,k}\lambda v_{v,a,k} + \sum_{\substack{k=1\\ i<k\\ j\neq k}}^{\mathrm{nS}}\theta_{i,j,k}\lambda \mathrm{v}_{v,a,i,k}\right. \\
& \left. + \sum_{\substack{k=1\\ k<i\\ j\neq k}}^{\mathrm{nS}}\theta_{i,j,k}\lambda \mathrm{v}_{v,a,k,i} + \sum_{\substack{k=1\\ j<k\\ i\neq k}}^{\mathrm{nS}}\theta_{i,j,k}\lambda \mathrm{v}_{v,a,j,k} + \sum_{\substack{k=1\\ k<j\\ i\neq k}}^{\mathrm{nS}}\theta_{i,j,k}\lambda \mathrm{v}_{v,a,k,j}\right)\mathcal{CC}_{v,a,i,j}
\end{aligned}
$$

$$
\begin{aligned}
\mathcal{CC}'_{w,a,i,j} = {} & (1-\delta_{1,a})(1-\phi w_{v,a})m_{a-1}\mathcal{CC}_{w,a-1,i,j} + (1-\delta_{1,a})\phi_{w,a}m_{a-1}\mathcal{CC}_{u,a-1,i,j} \\
& + (1-\delta_{1,a})\chi c_{w,a}m_{a-1}\mathcal{ZZ}_{v,a-1,i,j} + (1-\delta_{1,a})\xi c_{w,a}m_{a-1}\mathcal{ZZ}_{w,a-1,i,j} \\
& + (1-\delta_{1,a})\zeta c_{w,a}m_{a-1}\mathcal{ZZ}_{vw,a-1,i,j} + \psi_{w,a}\mathcal{CC}_{u,a,i,j} + \chi_{w,a}\mathcal{ZZ}_{v,a,i,j} + \xi_{w,a}\mathcal{ZZ}_{w,a,i,j} \\
& + \zeta_{w,a}\mathcal{ZZ}_{vw,a,i,j} + \theta_{i,j}(\lambda v_{w,a,j} + \lambda v_{w,a,i,j})\mathcal{C}_{w,a,i} + \theta_{j,i}(\lambda v_{w,a,i} + \lambda v_{w,a,i,j})\mathcal{C}_{w,a,j} \\
& + \lambda v_{w,a,i,j}\mathcal{N}_{w,a} + \sum_{\substack{k=1\\ i<j\\ j<k}}^{nS}\gamma_{w,a,k}\mathcal{CCC}_{w,a,i,j,k} + \sum_{\substack{k=1\\ i<k\\ k<j}}^{nS}\gamma_{w,a,k}\mathcal{CCC}_{w,a,i,k,j} \\
& + \sum_{\substack{k=1\\ k<i\\ i<j}}^{nS}\gamma_{w,a,k}\mathcal{CCC}_{w,a,k,i,j} \\
& - \left(\mu_a + m_a + \omega_{w,a} + \gamma_{w,a,i} + \gamma_{w,a,j} + \psi w_{v,a} + \sum_{\substack{k=1\\ k\neq i\\ k\neq j}}^{\mathrm{nS}}\theta_{i,j,k}\lambda v_{w,a,k} + \sum_{\substack{k=1\\ i<k\\ j\neq k}}^{\mathrm{nS}}\theta_{i,j,k}\lambda \mathrm{v}_{w,a,i,k}\right. \\
& \left. + \sum_{\substack{k=1\\ k<i\\ j\neq k}}^{\mathrm{nS}}\theta_{i,j,k}\lambda \mathrm{v}_{w,a,k,i} + \sum_{\substack{k=1\\ j<k\\ i\neq k}}^{\mathrm{nS}}\theta_{i,j,k}\lambda \mathrm{v}_{w,a,j,k} + \sum_{\substack{k=1\\ k<j\\ i\neq k}}^{\mathrm{nS}}\theta_{i,j,k}\lambda \mathrm{v}_{w,a,k,j}\right)\mathcal{CC}_{w,a,i,j}
\end{aligned}
$$

$$
\begin{aligned}
\mathcal{CC}'_{vw,a,i,j} = {} & (1-\delta_{1,a})m_{a-1}\mathcal{CC}_{vw,a-1,i,j} + (1-\delta_{1,a})\phi v_{w,a}m_{a-1}\mathcal{CC}_{v,a-1,i,j} \\
& + (1-\delta_{1,a})\phi w_{v,a}m_{a-1}\mathcal{CC}_{w,a-1,i,j} + \psi v_{w,a}\mathcal{CC}_{v,a,i,j} + \psi w_{v,a}\mathcal{CC}_{w,a,i,j} \\
& + \theta_{i,j}(\lambda v_{vw,a,j} + \lambda v_{vw,a,i,j})\mathcal{C}_{vw,a,i} + \theta_{j,i}(\lambda v_{vw,a,i} + \lambda v_{vw,a,i,j})\mathcal{C}_{vw,a,j} + \lambda v_{vw,a,i,j}\mathcal{N}_{vw,a} \\
& + \sum_{\substack{k=1\\ i<j\\ j<k}}^{nS}\gamma_{vw,a,k}\mathcal{CCC}_{vw,a,i,j,k} + \sum_{\substack{k=1\\ i<k\\ k<j}}^{nS}\gamma_{vw,a,k}\mathcal{CCC}_{vw,a,i,k,j} + \sum_{\substack{k=1\\ k<i\\ i<j}}^{nS}\gamma_{vw,a,k}\mathcal{CCC}_{vw,a,k,i,j} \\
& - \left(\mu_a + m_a + \omega_{vw,a} + \gamma_{vw,a,i} + \gamma_{vw,a,j} + \sum_{\substack{k=1\\ k\neq i\\ k\neq j}}^{\mathrm{nS}}\theta_{i,j,k}\lambda v_{vw,a,k} + \sum_{\substack{k=1\\ i<k\\ j\neq k}}^{\mathrm{nS}}\theta_{i,j,k}\lambda \mathrm{v}_{vw,a,i,k}\right. \\
& \left. + \sum_{\substack{k=1\\ k<i\\ j\neq k}}^{\mathrm{nS}}\theta_{i,j,k}\lambda \mathrm{v}_{vw,a,k,i} + \sum_{\substack{k=1\\ j<k\\ i\neq k}}^{\mathrm{nS}}\theta_{i,j,k}\lambda \mathrm{v}_{vw,a,j,k} + \sum_{\substack{k=1\\ k<j\\ i\neq k}}^{\mathrm{nS}}\theta_{i,j,k}\lambda \mathrm{v}_{vw,a,k,j}\right)\mathcal{CC}_{vw,a,i,j}.
\end{aligned}
$$

The age-specific population that was triple-colonized by STC i, j, and k was either unvaccinated ($\mathcal{CCC}_{u,a,i}$), vaccinated with a PCV ($\mathcal{CCC}_{v,a,i}$), vaccinated with a PPSV ($\mathcal{CCC}_{w,a,i}$), or vaccinated sequentially with both a PCV and PPSV ($\mathcal{CCC}_{vw,a,i}$) is represented by the following set of ODEs,

$$\begin{aligned}\mathcal{CCC}'_{u,a,i,j,k} = {} & (1-\delta_{1,a})(1-\phi_{v,a}-\phi_{w,a})m_{a-1}\mathcal{CCC}_{u,a-1,i,j} + \theta_{j,k,i}(\lambda_{a,i}+\lambda_{a,i,j}+\lambda_{a,i,k})\mathcal{CC}_{u,a,j,k} \\ & + \theta_{i,k,j}(\lambda_{a,j}+\lambda_{a,i,j}+\lambda_{a,j,k})\mathcal{CC}_{u,a,i,k} + \theta_{i,j,k}(\lambda_{a,k}+\lambda_{a,i,k}+\lambda_{a,j,k})\mathcal{CC}_{u,a,i,j} \\ & + \theta\theta_{i,j,k}\lambda_{a,j,k}\mathcal{C}_{u,a,i} + \theta\theta_{j,i,k}\lambda_{a,i,k}\mathcal{C}_{u,a,j} + \theta\theta_{k,i,j}\lambda_{a,i,j}\mathcal{C}_{u,a,k} \\ & - (\mu_a + m_a + \gamma_{u,a,i} + \gamma_{u,a,j} + \gamma_{u,a,k} + \psi_{v,a} + \psi_{w,a})\mathcal{CCC}_{u,a,i,j,k}\end{aligned}$$

$$\begin{aligned}\mathcal{CCC}'_{v,a,i,j,k} = {} & (1-\delta_{1,a})(1-\phi v_{w,a})m_{a-1}\mathcal{CCC}_{v,a-1,i,j,k} + (1-\delta_{1,a})\phi_{v,a}m_{a-1}\mathcal{CCC}_{u,a-1,i,j,k} \\ & + (1-\delta_{1,a})\chi c_{v,a}m_{a-1}\mathcal{ZZZ}_{v,a-1,i,j,k} + (1-\delta_{1,a})\xi c_{v,a}m_{a-1}\mathcal{ZZZ}_{w,a-1,i,j,k} \\ & + (1-\delta_{1,a})\zeta c_{v,a}m_{a-1}\mathcal{ZZZ}_{vw,a-1,i,j,k} + \psi_{v,a}\mathcal{CCC}_{u,a,i,j,k} + \chi_{v,a}\mathcal{ZZZ}_{v,a,i,j,k} \\ & + \xi_{v,a}\mathcal{ZZZ}_{w,a,i,j,k} + \zeta_{v,a}\mathcal{ZZZ}_{vw,a,i,j,k} + \theta_{j,k,i}(\lambda v_{v,a,i} + \lambda v_{v,a,i,j} + \lambda v_{v,a,i,k})\mathcal{CC}_{v,a,j,k} \\ & + \theta_{i,k,j}(\lambda v_{v,a,j} + \lambda v_{v,a,i,j} + \lambda v_{v,a,j,k})\mathcal{CC}_{v,a,i,k} \\ & + \theta_{i,j,k}(\lambda v_{v,a,k} + \lambda v_{v,a,i,k} + \lambda v_{v,a,j,k})\mathcal{CC}_{v,a,i,j} + \theta\theta_{i,j,k}\lambda v_{v,a,j,k}\mathcal{C}_{v,a,i} \\ & + \theta\theta_{j,i,k}\lambda v_{v,a,i,k}\mathcal{C}_{v,a,j} + \theta\theta_{k,i,j}\lambda v_{v,a,i,j}\mathcal{C}_{v,a,k} \\ & - (\mu_a + m_a + \gamma_{v,a,i} + \gamma_{v,a,j} + \gamma_{v,a,k} + \omega_{v,a} + \psi v_{w,a})\mathcal{CCC}_{v,a,i,j,k}\end{aligned}$$

$$\begin{aligned}\mathcal{CCC}'_{w,a,i,j,k} = {} & (1-\delta_{1,a})(1-\phi w_{v,a})m_{a-1}\mathcal{CCC}_{w,a-1,i,j,k} + (1-\delta_{1,a})\phi_{w,a}m_{a-1}\mathcal{CCC}_{u,a-1,i,j,k} \\ & + (1-\delta_{1,a})\chi c_{w,a}m_{a-1}\mathcal{ZZZ}_{v,a-1,i,j,k} + (1-\delta_{1,a})\xi c_{w,a}m_{a-1}\mathcal{ZZZ}_{w,a-1,i,j,k} \\ & + (1-\delta_{1,a})\zeta c_{w,a}m_{a-1}\mathcal{ZZZ}_{vw,a-1,i,j,k} + \psi_{w,a}\mathcal{CCC}_{u,a,i,j,k} + \chi_{w,a}\mathcal{ZZZ}_{v,a,i,j,k} \\ & + \xi_{w,a}\mathcal{ZZZ}_{w,a,i,j,k} + \zeta_{w,a}\mathcal{ZZZ}_{vw,a,i,j,k} + \theta_{j,k,i}(\lambda v_{w,a,i} + \lambda v_{w,a,i,j} + \lambda v_{w,a,i,k})\mathcal{CC}_{w,a,j,k} \\ & + \theta_{i,k,j}(\lambda v_{w,a,j} + \lambda v_{w,a,i,j} + \lambda v_{w,a,j,k})\mathcal{CC}_{w,a,i,k} \\ & + \theta_{i,j,k}(\lambda v_{w,a,k} + \lambda v_{w,a,i,k} + \lambda v_{w,a,j,k})\mathcal{CC}_{w,a,i,j} + \theta\theta_{i,j,k}\lambda v_{w,a,j,k}\mathcal{C}_{w,a,i} \\ & + \theta\theta_{j,i,k}\lambda v_{w,a,i,k}\mathcal{C}_{w,a,j} + \theta\theta_{k,i,j}\lambda v_{w,a,i,j}\mathcal{C}_{w,a,k} \\ & - (\mu_a + m_a + \gamma_{w,a,i} + \gamma_{w,a,j} + \gamma_{w,a,k} + \omega_{w,a} + \psi w_{v,a})\mathcal{CCC}_{w,a,i,j,k}\end{aligned}$$

$$\begin{aligned}\mathcal{CCC}'_{vw,a,i,j,k} = {} & (1-\delta_{1,a})m_{a-1}\mathcal{CCC}_{vw,a-1,i,j,k} + (1-\delta_{1,a})\phi v_{w,a}m_{a-1}\mathcal{CCC}_{v,a-1,i,j,k} \\ & + (1-\delta_{1,a})\phi w_{v,a}m_{a-1}\mathcal{CCC}_{w,a-1,i,j,k} + \psi v_{w,a}\mathcal{CCC}_{v,a,i,j,k} + \psi w_{v,a}\mathcal{CCC}_{w,a,i,j,k} \\ & + \theta_{j,k,i}(\lambda v_{vw,a,i} + \lambda v_{vw,a,i,j} + \lambda v_{vw,a,i,k})\mathcal{CC}_{vw,a,j,k} \\ & + \theta_{i,k,j}(\lambda v_{vw,a,j} + \lambda v_{vw,a,i,j} + \lambda v_{vw,a,j,k})\mathcal{CC}_{vw,a,i,k} \\ & + \theta_{i,j,k}(\lambda v_{vw,a,k} + \lambda v_{vw,a,i,k} + \lambda v_{vw,a,j,k})\mathcal{CC}_{vw,a,i,j} + \theta\theta_{i,j,k}\lambda v_{vw,a,j,k}\mathcal{C}_{vw,a,i} \\ & + \theta\theta_{j,i,k}\lambda v_{vw,a,i,k}\mathcal{C}_{vw,a,j} + \theta\theta_{k,i,j}\lambda v_{vw,a,i,j}\mathcal{C}_{vw,a,k} \\ & - (\mu_a + m_a + \gamma_{vw,a,i} + \gamma_{vw,a,j} + \gamma_{vw,a,k} + \omega_{vw,a})\mathcal{CCC}_{vw,a,i,j,k}\end{aligned}$$

The non-colonized, age-specific population that had waned vaccine protection from either PCV, PPSV, or a sequential PCV and PPSV was denoted by compartments $\mathcal{Z}_{v,a}$, $\mathcal{Z}_{w,a}$, or $\mathcal{Z}_{vw,a}$, and is represented by the following set of ODEs,

$$\begin{aligned}\mathcal{Z}'_{v,a} = {} & (1-\delta_{1,a})(1-\chi c_{v,a}-\chi c_{w,a})m_{a-1}\mathcal{Z}_{v,a-1} + \sum_{i=1}^{nS}\gamma_{u,a,i}\mathcal{Z}_{v,a,i} + \omega_{v,a}\mathcal{N}_{v,a} \\ & - \left(\mu_a + m_a + \chi_{v,a} + \chi_{w,a} + \sum_{i=1}^{nS}\lambda_{a,i} + \sum_{i=1}^{nS-1}\sum_{j=i+1}^{nS}\lambda_{a,i,j}\right)\mathcal{Z}_{v,a}\end{aligned}$$

$$\mathcal{Z}'_{w,a} = (1-\delta_{1,a})(1-\xi c_{v,a}-\xi c_{w,a})m_{a-1}\mathcal{Z}_{w,a-1} + \sum_{i=1}^{nS}\gamma_{u,a,i}\mathcal{Z}_{w,a,i} + \omega_{w,a}\mathcal{N}_{w,a} - \left(\mu_a + m_a + \xi_{v,a} + \xi_{w,a} + \sum_{i=1}^{nS}\lambda_{a,i} + \sum_{i=1}^{nS-1}\sum_{j=i+1}^{nS}\lambda_{a,i,j}\right)\mathcal{Z}_{w,a}$$

$$\mathcal{Z}'_{vw,a} = (1-\delta_{1,a})(1-\zeta c_{v,a}-\zeta c_{w,a})m_{a-1}\mathcal{Z}_{vw,a-1} + \sum_{i=1}^{nS}\gamma_{u,a,i}\mathcal{Z}_{vw,a,i} + \omega_{vw,a}\mathcal{N}_{vw,a} - \left(\mu_a + m_a + \zeta_{v,a} + \zeta_{w,a} + \sum_{i=1}^{nS}\lambda_{a,i} + \sum_{i=1}^{nS-1}\sum_{j=i+1}^{nS}\lambda_{a,i,j}\right)\mathcal{Z}_{vw,a}.$$

The age-specific population that was colonized by STC i and that had waned vaccine protection from either PCV, PPSV, or a sequential PCV and PPSV was denoted by compartments $\mathcal{Z}_{v,a,i}$, $\mathcal{Z}_{w,a,i}$, or $\mathcal{Z}_{vw,a,i}$, and is represented by the following set of ODEs,

$$\mathcal{Z}'_{v,a,i} = (1-\delta_{1,a})(1-\chi c_{v,a}-\chi c_{w,a})m_{a-1}\mathcal{Z}_{v,a-1,i} + \lambda_{a,i}\mathcal{Z}_{v,a} + \sum_{j=i+1}^{\mathrm{nS}}\gamma_{u,a,j}\mathcal{ZZ}_{v,a,i,j} + \sum_{j=1}^{\mathrm{i-1}}\gamma_{u,a,j}\mathcal{ZZ}_{v,a,j,i} + \omega_{v,a}\mathcal{C}_{v,a,i} - \left(\mu_a + m_a + \chi_{v,a} + \chi_{w,a} + \gamma_{u,a,i} + \sum_{\substack{j=1,\\ j\neq i}}^{\mathrm{nS}}\theta_{i,j}\lambda_{a,j} + \sum_{j=i+1}^{\mathrm{nS}}\theta_{i,j}\lambda_{a,i,j} + \sum_{j=1}^{\mathrm{i-1}}\theta_{i,j}\lambda_{a,j,i} + \sum_{\substack{j=1\\ j\neq i}}^{nS-1}\sum_{\substack{k=j+1\\ k\neq i}}^{nS}\theta\theta_{i,j,k}\lambda_{a,j,k}\right)\mathcal{Z}_{v,a,i}$$

$$\mathcal{Z}'_{w,a,i} = (1-\delta_{1,a})(1-\xi c_{v,a}-\xi c_{w,a})m_{a-1}\mathcal{Z}_{w,a-1,i} + \lambda_{a,i}\mathcal{Z}_{w,a} + \sum_{j=i+1}^{\mathrm{nS}}\gamma_{u,a,j}\mathcal{ZZ}_{w,a,i,j} + \sum_{j=1}^{\mathrm{i-1}}\gamma_{u,a,j}\mathcal{ZZ}_{w,a,j,i} + \omega_{w,a}\mathcal{C}_{w,a,i} - \left(\mu_a + m_a + \xi_{v,a} + \xi_{w,a} + \gamma_{u,a,i} + \sum_{\substack{j=1,\\ j\neq i}}^{\mathrm{nS}}\theta_{i,j}\lambda_{a,j} + \sum_{j=i+1}^{\mathrm{nS}}\theta_{i,j}\lambda_{a,i,j} + \sum_{j=1}^{\mathrm{i-1}}\theta_{i,j}\lambda_{a,j,i} + \sum_{\substack{j=1\\ j\neq i}}^{nS-1}\sum_{\substack{k=j+1\\ k\neq i}}^{nS}\theta\theta_{i,j,k}\lambda_{a,j,k}\right)\mathcal{Z}_{w,a,i}$$

$$\mathcal{Z}'_{vw,a,i} = (1-\delta_{1,a})(1-\zeta c_{v,a}-\zeta c_{w,a})m_{a-1}\mathcal{Z}_{vw,a-1,i} + \lambda_{a,i}\mathcal{Z}_{vw,a} + \sum_{j=i+1}^{\mathrm{nS}} \gamma_{u,a,j}\mathcal{Z}\mathcal{Z}_{vw,a,i,j} + \sum_{j=1}^{\mathrm{i-1}} \gamma_{u,a,j}\mathcal{Z}\mathcal{Z}_{vw,a,j,i} + \omega_{vw,a}\mathcal{C}_{vw,a,i} - \left(\mu_a + m_a + \zeta_{v,a} + \zeta_{w,a} + \gamma_{u,a,i} + \sum_{\substack{j=1,\\ j\neq i}}^{\mathrm{nS}} \theta_{i,j}\lambda_{a,j} + \sum_{j=i+1}^{\mathrm{nS}} \theta_{i,j}\lambda_{a,i,j} + \sum_{j=1}^{\mathrm{i-1}} \theta_{i,j}\lambda_{a,j,i} + \sum_{\substack{j=1\\ j\neq i}}^{nS-1} \sum_{\substack{k=j+1\\ k\neq i}}^{nS} \theta\theta_{i,j,k}\lambda_{a,j,k}\right)\mathcal{Z}_{vw,a,i}.$$

The age-specific population that was co-colonized by STC i and j and that had waned vaccine protection from either PCV, PPSV, or a sequential PCV and PPSV was denoted by compartments $\mathcal{Z}\mathcal{Z}_{v,a,i,j}$, $\mathcal{Z}\mathcal{Z}_{w,a,i,j}$, or $\mathcal{Z}\mathcal{Z}_{vw,a,i,j}$, and is represented by the following set of ODEs,

$$\mathcal{Z}\mathcal{Z}'_{v,a,i,j} = (1-\delta_{1,a})(1-\chi c_{v,a}-\chi c_{w,a})m_{a-1}\mathcal{Z}\mathcal{Z}_{v,a-1,i,j} + \theta_{i,j}(\lambda_{a,j}+\lambda_{a,i,j})\mathcal{Z}_{v,a,i} + \theta_{j,i}(\lambda_{a,i}+\lambda_{a,i,j})\mathcal{Z}_{v,a,j} + \omega_{v,a}\mathcal{C}\mathcal{C}_{v,a,i,j} + \lambda_{a,i,j}\mathcal{Z}_{v,a} + \sum_{\substack{k=1\\ i<j\\ j<k}}^{nS} \gamma_{u,a,k}\mathcal{Z}\mathcal{Z}\mathcal{Z}_{v,a,i,j,k} + \sum_{\substack{k=1\\ i<k\\ k<j}}^{nS} \gamma_{u,a,k}\mathcal{Z}\mathcal{Z}\mathcal{Z}_{v,a,i,k,j} + \sum_{\substack{k=1\\ k<i\\ i<j}}^{nS} \gamma_{u,a,k}\mathcal{Z}\mathcal{Z}\mathcal{Z}_{v,a,k,i,j} - \left(\mu_a + m_a + \gamma_{u,a,i} + \gamma_{u,a,j} + \chi_{v,a} + \chi_{w,a} + \sum_{\substack{k=1\\ k\neq i\\ k\neq j}}^{\mathrm{nS}} \theta_{i,j,k}\lambda_{a,k} + \sum_{\substack{k=1\\ i<k\\ j\neq k}}^{\mathrm{nS}} \theta_{i,j,k}\lambda_{a,i,k} + \sum_{\substack{k=1\\ k<i\\ j\neq k}}^{\mathrm{nS}} \theta_{i,j,k}\lambda_{a,k,i} + \sum_{\substack{k=1\\ j<k\\ i\neq k}}^{\mathrm{nS}} \theta_{i,j,k}\lambda_{a,j,k} + \sum_{\substack{k=1\\ k<j\\ i\neq k}}^{\mathrm{nS}} \theta_{i,j,k}\lambda_{a,k,j}\right)\mathcal{Z}\mathcal{Z}_{v,a,i,j}$$

$$\mathcal{ZZ}'_{w,a,i,j} = (1-\delta_{1,a})(1-\xi c_{v,a}-\xi c_{w,a})m_{a-1}\mathcal{ZZ}_{w,a-1,i,j} + \theta_{i,j}(\lambda_{a,j}+\lambda_{a,i,j})\mathcal{Z}_{w,a,i} + \theta_{j,i}(\lambda_{a,i}+\lambda_{a,i,j})\mathcal{Z}_{w,a,j} + \omega_{w,a}\mathcal{CC}_{w,a,i,j} + \lambda_{a,i,j}\mathcal{Z}_{w,a} + \sum_{\substack{k=1\\i<j\\j<k}}^{nS}\gamma_{u,a,k}\mathcal{ZZZ}_{w,a,i,j,k} + \sum_{\substack{k=1\\i<k\\k<j}}^{nS}\gamma_{u,a,k}\mathcal{ZZZ}_{w,a,i,k,j} + \sum_{\substack{k=1\\k<i\\i<j}}^{nS}\gamma_{u,a,k}\mathcal{ZZZ}_{w,a,k,i,j} - \left(\mu_a + m_a + \gamma_{u,a,i} + \gamma_{u,a,j} + \xi_{v,a} + \xi_{w,a} + \sum_{\substack{k=1\\k\neq i\\k\neq j}}^{\mathrm{nS}}\theta_{i,j,k}\lambda_{a,k} + \sum_{\substack{k=1\\i<k\\j\neq k}}^{\mathrm{nS}}\theta_{i,j,k}\lambda_{a,i,k} + \sum_{\substack{k=1\\k<i\\j\neq k}}^{\mathrm{nS}}\theta_{i,j,k}\lambda_{a,k,i} + \sum_{\substack{k=1\\j<k\\i\neq k}}^{\mathrm{nS}}\theta_{i,j,k}\lambda_{a,j,k} + \sum_{\substack{k=1\\k<j\\i\neq k}}^{\mathrm{nS}}\theta_{i,j,k}\lambda_{a,k,j}\right)\mathcal{ZZ}_{w,a,i,j}$$

$$\mathcal{ZZ}'_{vw,a,i,j} = (1-\delta_{1,a})(1-\zeta c_{v,a}-\zeta c_{w,a})m_{a-1}\mathcal{ZZ}_{vw,a-1,i,j} + \theta_{i,j}(\lambda_{a,j}+\lambda_{a,i,j})\mathcal{Z}_{vw,a,i} + \theta_{j,i}(\lambda_{a,i}+\lambda_{a,i,j})\mathcal{Z}_{vw,a,j} + \omega_{vw,a}\mathcal{CC}_{vw,a,i,j} + \lambda_{a,i,j}\mathcal{Z}_{vw,a} + \sum_{\substack{k=1\\i<j\\j<k}}^{nS}\gamma_{u,a,k}\mathcal{ZZZ}_{vw,a,i,j,k} + \sum_{\substack{k=1\\i<k\\k<j}}^{nS}\gamma_{u,a,k}\mathcal{ZZZ}_{vw,a,i,k,j} + \sum_{\substack{k=1\\k<i\\i<j}}^{nS}\gamma_{u,a,k}\mathcal{ZZZ}_{vw,a,k,i,j} - \left(\mu_a + m_a + \gamma_{u,a,i} + \gamma_{u,a,j} + \zeta_{v,a} + \zeta_{w,a} + \sum_{\substack{k=1\\k\neq i\\k\neq j}}^{\mathrm{nS}}\theta_{i,j,k}\lambda_{a,k} + \sum_{\substack{k=1\\i<k\\j\neq k}}^{\mathrm{nS}}\theta_{i,j,k}\lambda_{a,i,k} + \sum_{\substack{k=1\\k<i\\j\neq k}}^{\mathrm{nS}}\theta_{i,j,k}\lambda_{a,k,i} + \sum_{\substack{k=1\\j<k\\i\neq k}}^{\mathrm{nS}}\theta_{i,j,k}\lambda_{a,j,k} + \sum_{\substack{k=1\\k<j\\i\neq k}}^{\mathrm{nS}}\theta_{i,j,k}\lambda_{a,k,j}\right)\mathcal{ZZ}_{vw,a,i,j}.$$

The age-specific population that was triple-colonized by STC i, j, and k and that had waned vaccine protection from either PCV, PPSV, or a sequential PCV and PPSV was denoted by compartments $\mathcal{ZZZ}_{v,a,i,j,k}$, $\mathcal{ZZZ}_{w,a,i,j,k}$, or $\mathcal{ZZZ}_{vw,a,i,j,k}$, and is represented by the following set of ODEs,

$$
\begin{aligned}
ZZZ'_{v,a,i,j,k} = {} & (1-\delta_{1,a})(1-\chi c_{v,a}-\chi c_{w,a})m_{a-1}ZZZ_{v,a-1,i,j} + \omega_{v,a}CCC_{v,a,i,j,k} \\
& + \theta_{j,k,i}(\lambda_{a,i}+\lambda_{a,i,j}+\lambda_{a,i,k})ZZ_{v,a,j,k} + \theta_{i,k,j}(\lambda_{a,j}+\lambda_{a,i,j}+\lambda_{a,j,k})ZZ_{v,a,i,k} \\
& + \theta_{i,j,k}(\lambda_{a,k}+\lambda_{a,i,k}+\lambda_{a,j,k})ZZ_{v,a,i,j} + \theta\theta_{i,j,k}\lambda_{a,j,k}Z_{v,a,i} + \theta\theta_{j,i,k}\lambda_{a,i,k}Z_{v,a,j} \\
& + \theta\theta_{k,i,j}\lambda_{a,i,j}Z_{v,a,k} - (\mu_a + m_a + \gamma_{u,a,i} + \gamma_{u,a,j} + \gamma_{u,a,k} + \chi_{v,a} + \chi_{w,a})ZZZ_{v,a,i,j,k}
\end{aligned}
$$

$$
\begin{aligned}
ZZZ'_{w,a,i,j,k} = {} & (1-\delta_{1,a})(1-\xi c_{v,a}-\xi c_{w,a})m_{a-1}ZZZ_{w,a-1,i,j} + \omega_{w,a}CCC_{w,a,i,j,k} \\
& + \theta_{j,k,i}(\lambda_{a,i}+\lambda_{a,i,j}+\lambda_{a,i,k})ZZ_{w,a,j,k} + \theta_{i,k,j}(\lambda_{a,j}+\lambda_{a,i,j}+\lambda_{a,j,k})ZZ_{w,a,i,k} \\
& + \theta_{i,j,k}(\lambda_{a,k}+\lambda_{a,i,k}+\lambda_{a,j,k})ZZ_{w,a,i,j} + \theta\theta_{i,j,k}\lambda_{a,j,k}Z_{w,a,i} + \theta\theta_{j,i,k}\lambda_{a,i,k}Z_{w,a,j} \\
& + \theta\theta_{k,i,j}\lambda_{a,i,j}Z_{w,a,k} - (\mu_a + m_a + \gamma_{u,a,i} + \gamma_{u,a,j} + \gamma_{u,a,k} + \xi_{v,a} + \xi_{w,a})ZZZ_{w,a,i,j,k}
\end{aligned}
$$

$$
\begin{aligned}
ZZZ'_{vw,a,i,j,k} = {} & (1-\delta_{1,a})(1-\zeta c_{v,a}-\zeta c_{w,a})m_{a-1}ZZZ_{vw,a-1,i,j} + \omega_{vw,a}CCC_{vw,a,i,j,k} \\
& + \theta_{j,k,i}(\lambda_{a,i}+\lambda_{a,i,j}+\lambda_{a,i,k})ZZ_{vw,a,j,k} + \theta_{i,k,j}(\lambda_{a,j}+\lambda_{a,i,j}+\lambda_{a,j,k})ZZ_{vw,a,i,k} \\
& + \theta_{i,j,k}(\lambda_{a,k}+\lambda_{a,i,k}+\lambda_{a,j,k})ZZ_{vw,a,i,j} + \theta\theta_{i,j,k}\lambda_{a,j,k}Z_{vw,a,i} + \theta\theta_{j,i,k}\lambda_{a,i,k}Z_{vw,a,j} \\
& + \theta\theta_{k,i,j}\lambda_{a,i,j}Z_{vw,a,k} - (\mu_a + m_a + \gamma_{u,a,i} + \gamma_{u,a,j} + \gamma_{u,a,k} + \zeta_{v,a} + \zeta_{w,a})ZZZ_{vw,a,i,j,k}
\end{aligned}
$$

Model Calibration

Calibrated Parameters

Table S3. Calibrated probability of acquisition of carriage by age group and serotype ($\beta_{a,i}$).

Age group (yrs)	Serotype class:										
	1	2	3	4	5	6	7	8	9	10	11
<2	0.0113	0.0229	0.0079	0.0112	0.0074	0.0059	0.0121	0.0117	0.0102	0.0160	0.0123
2-5	0.0154	0.0162	0.0193	0.0061	0.0049	0.0058	0.0708	0.0110	0.0312	0.0316	0.0291
5-18	0.0127	0.0366	0.0158	0.0138	0.0160	0.0057	0.0124	0.0122	0.0099	0.0144	0.0116
18-50	0.0022	0.0216	0.0028	0.0068	0.0033	0.0031	0.0068	0.0062	0.0114	0.0062	0.0042
50-60	0.0052	0.0074	0.0022	0.0022	0.0020	0.0032	0.0081	0.0026	0.0070	0.0057	0.0022
60+	0.0200	0.0273	0.0103	0.0103	0.0039	0.0113	0.0284	0.0304	0.0296	0.0219	0.0094

Table S4. Calibrated competition parameter by serotype ($\theta_{i,j}$)

Serotype class	1	2	3	4	5	6	7	8	9	10	11
1	0	0.72	0.46	0.36	0.4	0.63	0.36	0.74	0.81	0.27	0.7
2	1	0	1	1	1	0.34	0.09	0.7	0.81	0.09	0.54
3	1	1	0	1	1	1	1	1	1	1	1
4	1	1	1	0	1	0.54	0.09	0.72	0.81	0.09	0.54
5	1	1	1	1	0	0.54	0.09	0.72	0.81	0.09	0.54
6	1	1	1	1	1	0	1	1	1	1	1
7	1	1	1	1	1	1	0	1	1	1	1
8	1	1	1	1	1	1	1	0	1	1	1

9	1	1	1	1	1	1	1	1	0	1	1
10	1	1	1	1	1	1	1	1	1	0	1
11	1	1	1	1	1	1	1	1	1	1	0

Table S5. Probability of developing IPD given carriage by age group and serotype ($\rho_{a,i}$)

Age group (yrs)	Serotype class:										
	1	2	3	4	5	6	7	8	9	10	11
<2	0.00005	0.000036	0.00005	0.000149	0.000025	0.000014	0.000004	0.000071	0.000013	0.000015	0.000028
2-5	0.000012	0.000052	0.000015	0.000084	0.000013	0.000005	0	0.000023	0.000002	0.000004	0.000003
5-18	0.000006	0.000009	0.000003	0.000032	0.000001	0.000003	0.000002	0.00002	0.000004	0.000005	0.00001
18-50	0.000013	0.000001	0.000004	0.000005	0.000002	0.000001	0.000002	0.00001	0	0.000003	0.000003
50-60	0.000025	0.000006	0.000043	0.000071	0.000021	0.000009	0.000008	0.000127	0.000002	0.000023	0.000033
60+	0.000017	0.000005	0.000049	0.000082	0.000096	0.000013	0.00001	0.000026	0.000002	0.000026	0.00004

Table S6. Probability of developing inpatient NBPP given carriage by age group and serotype ($\rho_{a,i}$)

Age group (yrs)	Serotype class:										
	1	2	3	4	5	6	7	8	9	10	11
<2	0.000547	0.000717	0.003973	0.004041	0.000364	0.000644	0.000163	0.00316	0.000452	0.000667	0.00124
2-5	0.000394	0.001903	0.002285	0.009512	0.00123	0.000576	0.000042	0.002438	0.000223	0.000416	0.000256
5-18	0.000286	0.000204	0.000191	0.002504	0.000067	0.000219	0.000191	0.001781	0.000228	0.000379	0.0008
18-50	0.000439	0.000001	0.00009	0.000061	0.000028	0.00002	0.00005	0.000266	0.000003	0.000069	0.00007
50-60	0.000543	0.000007	0.00069	0.000788	0.000273	0.000123	0.000154	0.002109	0.000025	0.000344	0.000549
60+	0.000796	0.000019	0.002541	0.003678	0.004202	0.000406	0.000663	0.001479	0.000127	0.001568	0.002621

Table S7. Probability of developing outpatient NBPP given carriage by age group and serotype ($\rho_{a,i}$)

Age group (yrs)	Serotype class:										
	1	2	3	4	5	6	7	8	9	10	11
<2	0.000775	0.001015	0.005622	0.005718	0.000514	0.000911	0.00023	0.004472	0.00064	0.000943	0.001755
2-5	0.000557	0.002694	0.003233	0.013461	0.001741	0.000815	0.000059	0.00345	0.000315	0.000589	0.000363
5-18	0.000405	0.000288	0.000271	0.003544	0.000095	0.000309	0.00027	0.00252	0.000322	0.000537	0.001132
18-50	0.001666	0.000005	0.000342	0.000232	0.000105	0.000076	0.000189	0.001007	0.000013	0.000262	0.000267
50-60	0.001044	0.000014	0.001325	0.001513	0.000525	0.000236	0.000296	0.004052	0.000048	0.000662	0.001054
60+	0.000434	0.000011	0.001387	0.002007	0.002293	0.000221	0.000362	0.000807	0.000069	0.000856	0.00143

Table S8. Vaccine efficacy against carriage acquisition by age group and serotype class ($\varepsilon_{a,i}$)

Age group	Serotype class:				
	1	2	3	4	5

Pediatric	0.49	0.1274	0.01	0.3	0.1
Adult	0.3	0.0531	0.005	0.25	0.05

Model Fits

Figure S2. Model calibration fit results for IPD by age group and serotype.

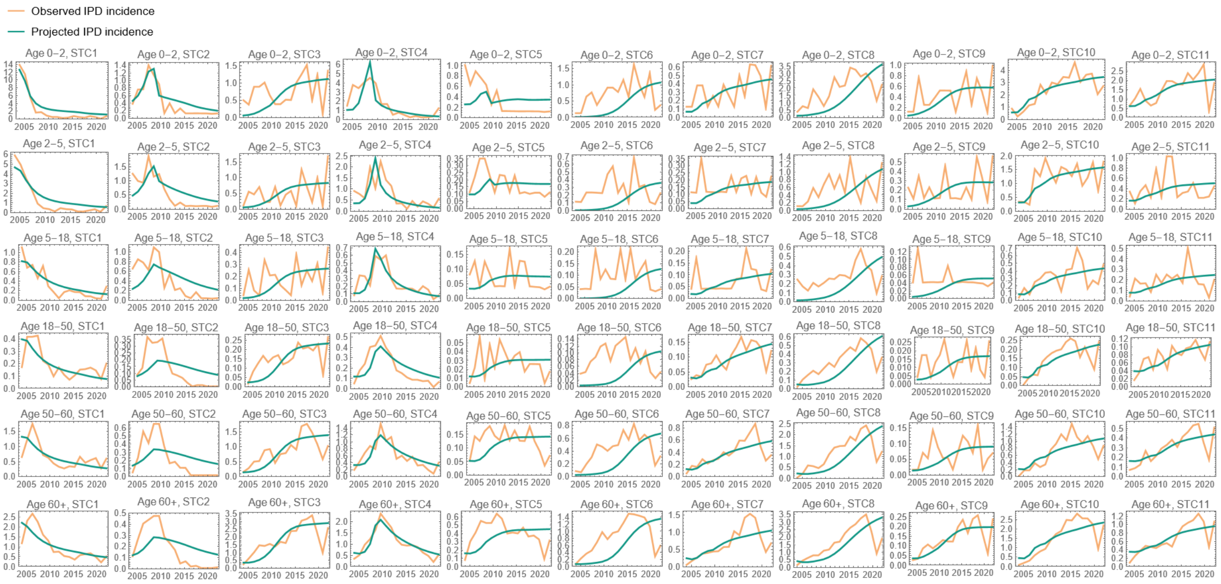

Figure S3. Model calibration fit results for inpatient NBPP by age group and serotype.

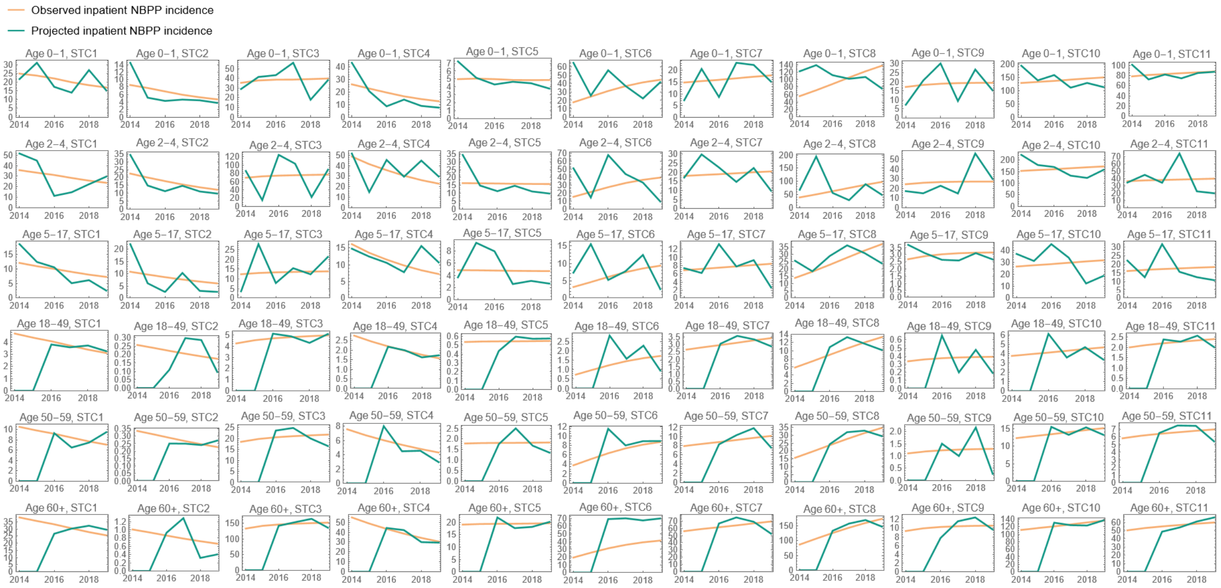

Figure S4. Model calibration fit results for outpatient NBPP by age group and serotype.

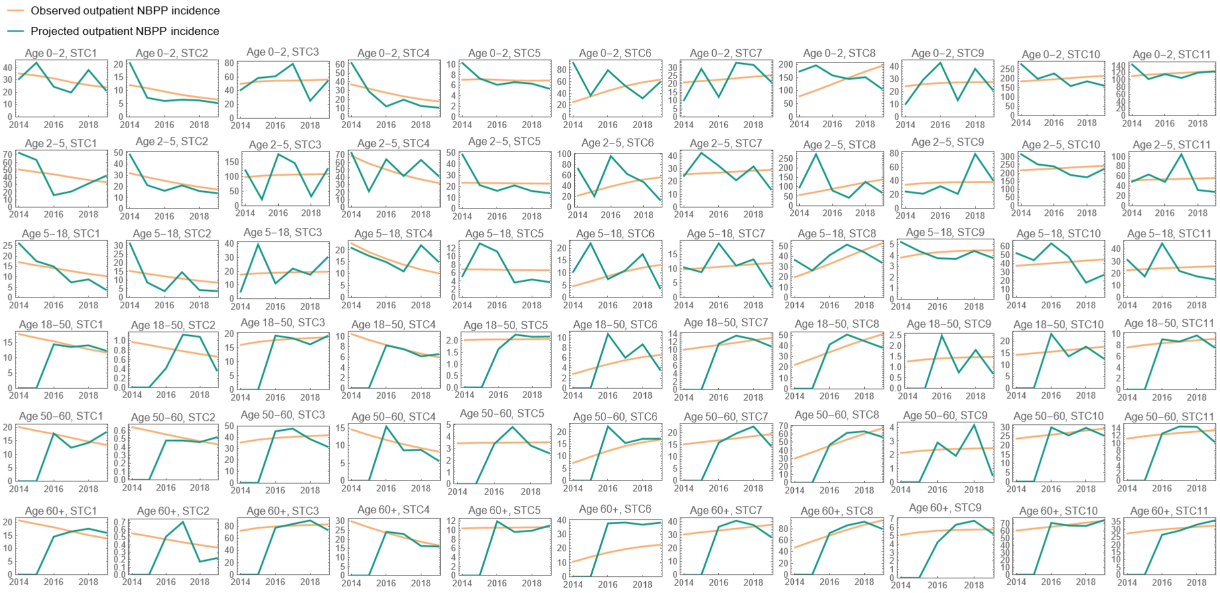

Vaccine Efficacy

IPD

Table S9. Vaccine efficacies against IPD for the 0-1-year-old age group for PCV7, PCV10, PCV13, and PCV15 [2-5].

Serotype class (STs)	VE against IPD (PCV7)	VE against IPD (PCV13)	VE against IPD (PCV15)
1 (4, 6B, 9V, 14, 18C, 19F, 23F)	0.947	0.947	0.947
2 (1, 5)	0	0.87	0.87
3 (3)	0	0.3	0.3
4 (7F, 19A)	0	0.915	0.915
5 (6A, 6C)	0	0.86	0.86
6 (22F, 33F)	0	0	0.86
7 (9N, 17F, 20)	0	0	0
8 (8, 10A, 11A, 12F)	0	0	0
9 (15B)	0	0	0
10 (15A, 15C, 16F, 23A, 23B, 24F, 31, 35B)	0	0	0
11 (NVTs)	0	0	0

Table S10. Vaccine efficacies against IPD for the 15-49- and 50-64-year-old risk group and 65+ year-old population for PPSV23 [6,7], V116, and PCV20 [4,8].

Serotype class (STs)	VE against IPD (PPSV23)	VE against IPD (V116)	VE against IPD (PCV20)
1 (4, 6B, 9V, 14, 18C, 19F, 23F)	0.597	0	0.75
2 (1, 5)	0.597	0	0.75
3 (3)	0.597	0.26	0.26
4 (7F, 19A)	0.597	0.75	0.75
5 (6A, 6C)	0	0.75	0.75
6 (22F, 33F)	0.597	0.75	0.75
7 (9N, 17F, 20)	0.597	0.75	0
8 (8, 10A, 11A, 12F)	0.597	0.75	0.75
9 (15B)	0.597	0.75	0.75
10 (15A, 15C, 16F, 23A, 23B, 24F, 31, 35B)	0	0.75	0
11 (NVTs)	0	0	0

NBPP

Table S11. Vaccine efficacies against NBPP for 0-1-year-old age group for PCV7, PCV10, PCV13, and PCV15 [9].

Serotype class (STs)	VE against NBPP (PCV7)	VE against NBPP (PCV13)	VE against NBPP (PCV15)
1 (4, 6B, 9V, 14, 18C, 19F, 23F)	0.87	0.87	0.87
2 (1, 5)	0	0.87	0.87
3 (3)	0	0.87	0.87
4 (7F, 19A)	0	0.87	0.87
5 (6A, 6C)	0	0.87	0.87
6 (22F, 33F)	0	0	0.87
7 (9N, 17F, 20)	0	0	0
8 (8, 10A, 11A, 12F)	0	0	0
9 (15B)	0	0	0

10 (15A, 15C, 16F, 23A, 23B, 24F, 31, 35B)	0	0	0
10 (NVTs)	0	0	0

Table S12. Vaccine efficacies against NBPP for the 15-49- and 50-64-year-old risk group and 65+ year-old population for PPSV23 [7].

Serotype class (STs)	VE against NBPP (PPSV23)
1 (4, 6B, 9V, 14, 18C, 19F, 23F)	0.34
2 (1, 5)	0.34
3 (3)	0.34
4 (7F, 19A)	0.34
5 (6A, 6C)	0
6 (22F, 33F)	0.34
7 (9N, 17F, 20)	0.34
8 (8, 10A, 11A, 12F)	0.34
9 (15B)	0.34
10 (15A, 15C, 16F, 23A, 23B, 24F, 31, 35B)	0
11 (NVTs)	0

Table S13. Vaccine efficacies against NBPP for the 15-49- and 50-64-year-old risk group and 65+ year-old population for V116 and PCV20 [8].

Serotype class (STs)	VE against NBPP (15-49-year-olds)	VE against NBPP (50-64-year-olds)	VE against NBPP (65+ year-olds)
1 (4, 6B, 9V, 14, 18C, 19F, 23F)	0.556	0.513	0.45
2 (1, 5)	0.556	0.513	0.45
3 (3)	0.556	0.513	0.45
4 (7F, 19A)	0.556	0.513	0.45
5 (6A, 6C)	0.556	0.513	0.45
6 (22F, 33F)	0.556	0.513	0.45
7 (9N, 17F, 20)	0	0	0

8 (8, 10A, 11A, 12F)	0.556	0.513	0.45
9 (15B)	0.556	0.513	0.45
10 (15A, 15C, 16F, 23A, 23B, 24F, 31, 35B)	0	0	0
11 (NVTs)	0	0	0

Additional Model Results

IPD

Figure S5. Impact of adult vaccination scenarios on IPD incidence at the 10-year time horizon (cases per 100,000 population). Scenario 0: PPSV23 with 30% uptake (status quo); labels are percent change compared with scenario 0.

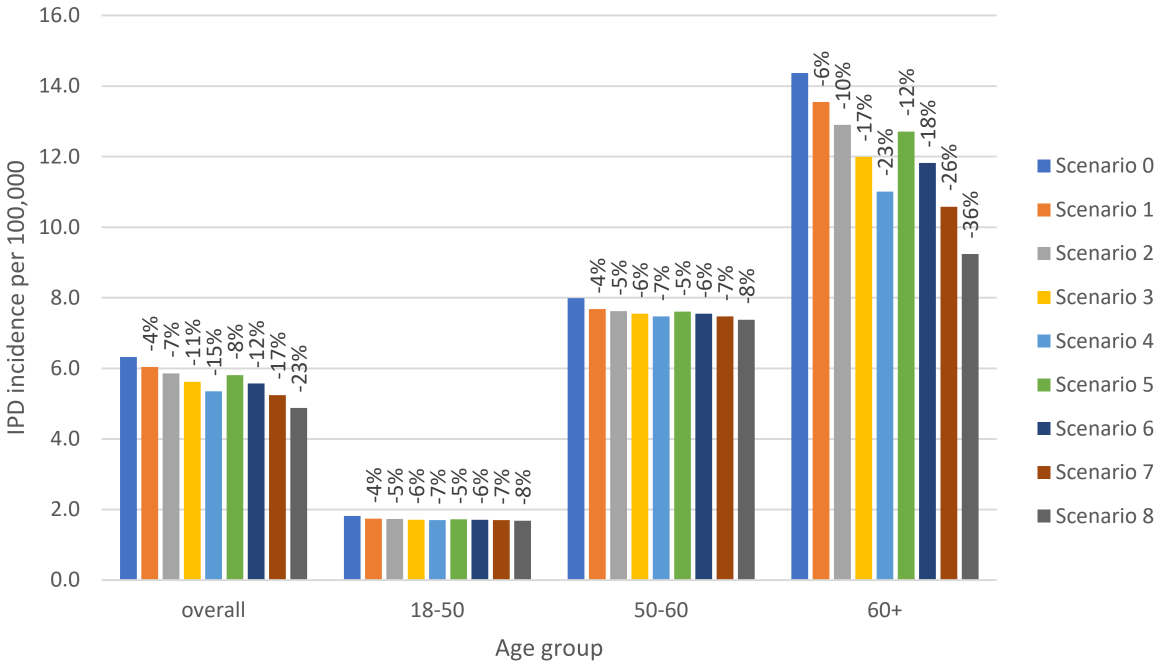

Table S14. Comparative impact of vaccine formulation, increased uptake, and revaccination on IPD incidence over 10-year time horizon (% change versus comparator scenario). Impact of vaccine: % change in outcomes for V116 scenarios as compared with equivalent (i.e., matching uptake and revaccination) PCV20 scenarios. Impact of uptake: % change in outcomes for scenarios with 60% uptake as compared with equivalent (i.e., matching vaccine and revaccination) scenarios with 30% uptake. Impact of revaccination: % change in outcomes for scenarios with revaccination as compared with equivalent (i.e., matching vaccine and uptake) scenarios without revaccination.

Scenario number	Impact of vaccine				Impact of uptake				Impact of revaccination			
	overall	18-50	50-60	60+	overall	18-50	50-60	60+	overall	18-50	50-60	60+

Scenario 1												
Scenario 2									-3.0%	-0.6%	-0.8%	-4.8%
Scenario 3					-7.0%	-1.7%	-1.7%	-11.5%				
Scenario 4					-8.7%	-1.7%	-2.0%	-14.7%	-4.8%	-0.6%	-1.1%	-8.2%
Scenario 5	-3.8%	-1.1%	-0.9%	-6.2%								
Scenario 6	-4.9%	-1.2%	-0.9%	-8.4%					-4.1%	-0.6%	-0.8%	-7.0%
Scenario 7	-6.8%	-0.6%	-1.1%	-11.8%	-9.8%	-1.2%	-1.8%	-16.8%				
Scenario 8	-8.8%	-1.2%	-1.2%	-16.1%	-12.4%	-1.8%	-2.3%	-21.8%	-6.9%	-1.2%	-1.2%	-12.7%

Table S15. Comparative impact of vaccine formulation, increased uptake, and revaccination on cumulative IPD cases over 10-year time horizon (% change versus comparator scenario). Impact of vaccine: % change in outcomes for V116 scenarios as compared with equivalent (i.e., matching uptake and revaccination) PCV20 scenarios. Impact of uptake: % change in outcomes for scenarios with 60% uptake as compared with equivalent (i.e., matching vaccine and revaccination) scenarios with 30% uptake. Impact of revaccination: % change in outcomes for scenarios with revaccination as compared with equivalent (i.e., matching vaccine and uptake) scenarios without revaccination.

Scenario number	Impact of vaccine				Impact of uptake				Impact of revaccination			
	overall	18-50	50-60	60+	overall	18-50	50-60	60+	overall	18-50	50-60	60+
Scenario 1												
Scenario 2									-3.2%	-0.6%	-0.7%	-5.4%
Scenario 3					-4.1%	-0.8%	-0.9%	-6.8%				
Scenario 4					-4.8%	-0.9%	-1.0%	-8.2%	-3.9%	-0.7%	-0.9%	-6.7%
Scenario 5	-3.2%	-0.6%	-0.6%	-5.3%								
Scenario 6	-4.4%	-0.7%	-0.7%	-7.5%					-4.4%	-0.7%	-0.8%	-7.6%
Scenario 7	-4.7%	-0.7%	-0.7%	-8.2%	-5.6%	-0.8%	-1.0%	-9.7%				
Scenario 8	-6.3%	-0.8%	-0.8%	-11.3%	-6.7%	-1.0%	-1.2%	-11.9%	-5.5%	-0.8%	-1.0%	-9.9%

Table S16. Distribution of cumulative IPD cases covered by PCV20 but not V116, and vice-versa (total cases).

Scenario number	Cumulative IPD PCV20 / non-V116			Cumulative IPD V116 / non-PCV20		
	18-50 10y	50-60 10y	60+ 10y	18-50 10y	50-60 10y	60+ 10y
Scenario 1	523	458	1,257	1,271	1,714	7,399
Scenario 2	520	452	1,105	1,272	1,716	7,420
Scenario 3	519	450	1,069	1,272	1,717	7,425
Scenario 4	515	444	898	1,274	1,719	7,449
Scenario 5	557	491	1,768	1,208	1,650	5,514
Scenario 6	558	491	1,770	1,204	1,642	4,905
Scenario 7	558	491	1,770	1,203	1,640	4,730
Scenario 8	558	492	1,772	1,199	1,630	3,998

Inpatient NBPP

Figure S6. Impact of adult vaccination scenarios on inpatient NBPP incidence at the 10-year time horizon (cases per 100,000 population). Scenario 0: PPSV23 with 30% uptake (status quo); labels are percent change compared with scenario 0.

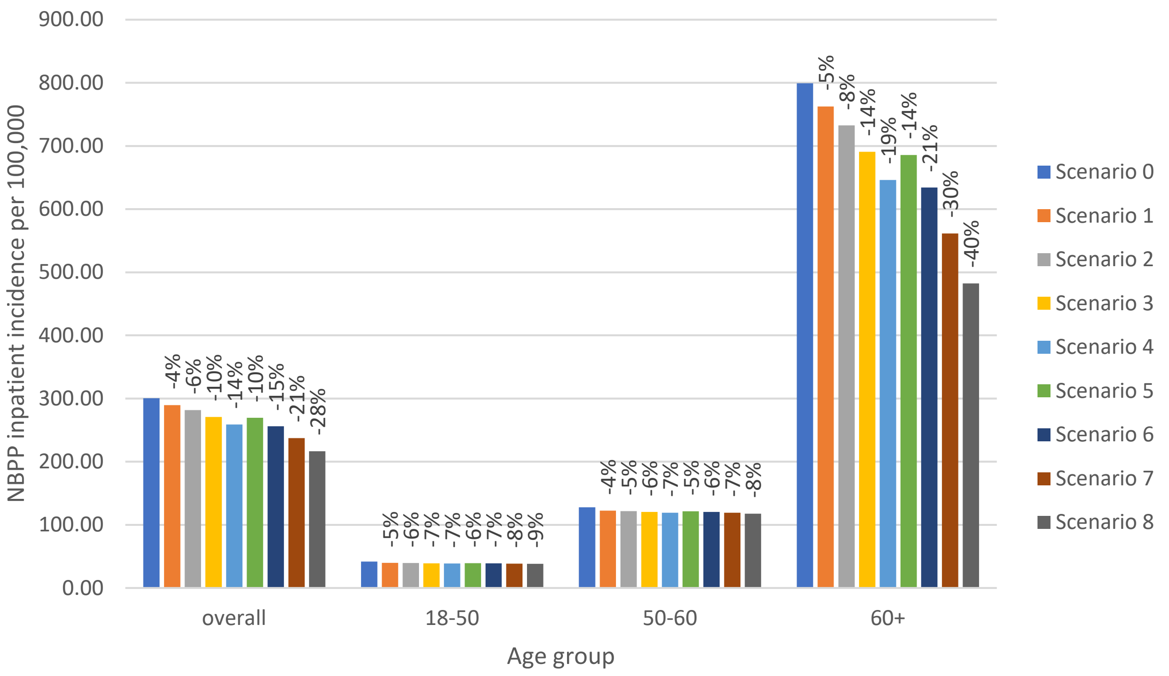

Table S17. Comparative impact of vaccine formulation, increased uptake, and revaccination on inpatient NBPP incidence over 10-year time horizon (% change versus comparator scenario). Impact of vaccine: % change in outcomes for V116 scenarios as compared with equivalent (i.e., matching uptake and revaccination) PCV20 scenarios. Impact of uptake: % change in outcomes for scenarios with 60% uptake as compared with equivalent (i.e., matching vaccine and revaccination) scenarios with 30% uptake. Impact of revaccination: % change in outcomes for scenarios with revaccination as compared with equivalent (i.e., matching vaccine and uptake) scenarios without revaccination.

Scenario number	Impact of vaccine				Impact of uptake				Impact of revaccination			
	overall	18-50	50-60	60+	overall	18-50	50-60	60+	overall	18-50	50-60	60+
Scenario 1												
Scenario 2									-2.7%	-0.7%	-0.7%	-3.9%
Scenario 3					-6.5%	-1.5%	-1.7%	-9.4%				
Scenario 4					-8.1%	-1.8%	-2.1%	-11.8%	-4.3%	-1.0%	-1.1%	-6.5%
Scenario 5	-6.9%	-1.2%	-0.9%	-10.1%								
Scenario 6	-9.1%	-1.2%	-1.0%	-13.4%					-5.0%	-0.7%	-0.8%	-7.5%
Scenario 7	-12.4%	-1.3%	-1.1%	-18.7%	-12.0%	-1.6%	-1.9%	-18.1%				
Scenario 8	-16.3%	-1.3%	-1.2%	-25.3%	-15.4%	-2.0%	-2.3%	-23.9%	-8.7%	-1.1%	-1.2%	-14.1%

Table S18. Comparative impact of vaccine formulation, increased uptake, and revaccination on cumulative inpatient NBPP cases over 10-year time horizon (% change versus comparator scenario). Impact of vaccine: % change in outcomes for V116 scenarios as compared with equivalent (i.e., matching uptake and revaccination) PCV20 scenarios. Impact of uptake: % change in outcomes for scenarios with 60% uptake as compared with equivalent (i.e., matching vaccine and revaccination) scenarios with 30% uptake. Impact of revaccination: % change in outcomes for scenarios with revaccination as compared with equivalent (i.e., matching vaccine and uptake) scenarios without revaccination.

Scenario number	Impact of vaccine				Impact of uptake				Impact of revaccination			
	overall	18-50	50-60	60+	overall	18-50	50-60	60+	overall	18-50	50-60	60+
Scenario 1												
Scenario 2									-3.0%	-0.7%	-0.8%	-4.3%
Scenario 3					-3.8%	-0.8%	-1.0%	-5.5%				
Scenario 4					-4.4%	-1.0%	-1.1%	-6.5%	-3.6%	-0.8%	-0.9%	-5.3%
Scenario 5	-6.0%	-0.9%	-0.7%	-8.9%								
Scenario 6	-8.3%	-0.9%	-0.7%	-12.4%					-5.3%	-0.7%	-0.8%	-8.1%
Scenario 7	-9.0%	-0.9%	-0.7%	-13.5%	-6.8%	-0.9%	-1.0%	-10.3%				
Scenario 8	-12.0%	-1.0%	-0.8%	-18.4%	-8.3%	-1.0%	-1.2%	-12.9%	-6.8%	-0.9%	-1.0%	-10.7%

Table S19. Distribution of cumulative inpatient NBPP cases covered by PCV20 but not V116, and vice-versa (total cases).

Scenario number	Cumulative IPD PCV20 / non-V116			Cumulative IPD V116 / non-PCV20		
	18-50 10y	50-60 10y	60+ 10y	18-50 10y	50-60 10y	60+ 10y
Scenario 1	9,408	6,698	54,428	29,631	27,777	496,053
Scenario 2	9,304	6,571	48,353	29,658	27,809	497,446
Scenario 3	9,282	6,545	46,881	29,663	27,815	497,756
Scenario 4	9,169	6,407	40,127	29,693	27,851	499,303
Scenario 5	10,131	7,296	75,154	28,225	26,731	384,854
Scenario 6	10,135	7,300	75,238	28,134	26,601	349,291
Scenario 7	10,135	7,301	75,257	28,108	26,565	339,143
Scenario 8	10,139	7,305	75,353	27,998	26,408	296,417

Outpatient NBPP

Figure S7. Impact of adult vaccination scenarios on outpatient NBPP incidence over 10-year time horizon (cases per 100,000 population). Scenario 0: PPSV23 with 30% uptake (status quo); labels are percent change compared with scenario 0.

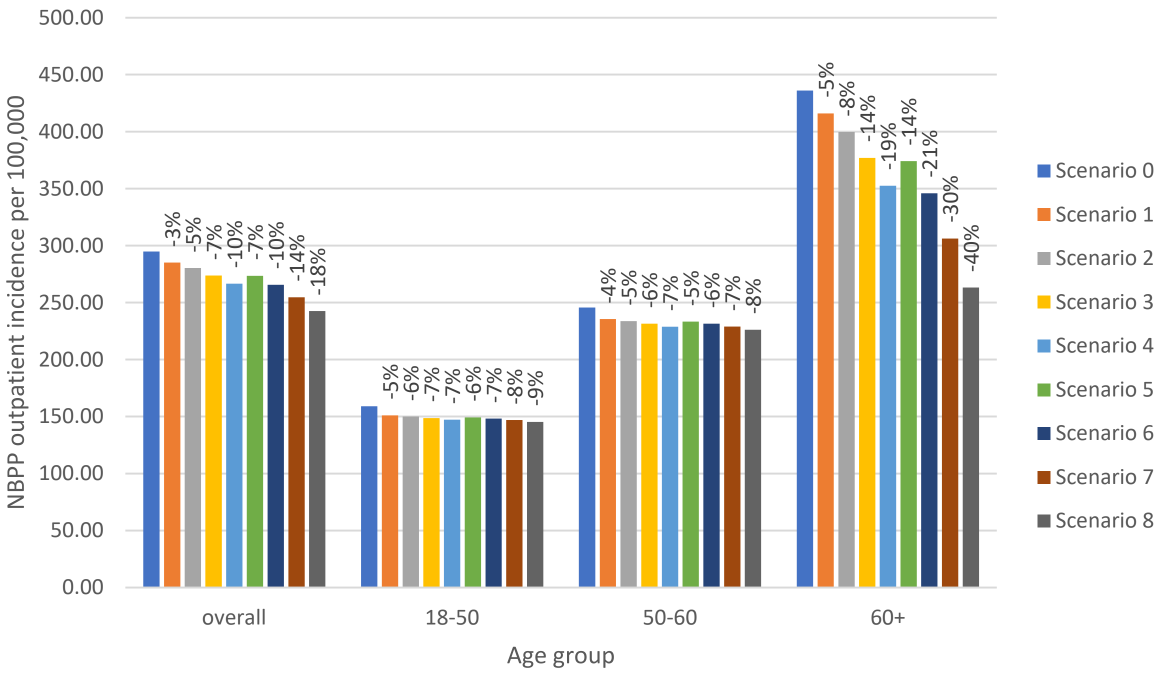

Table S20. Comparative impact of vaccine formulation, increased uptake, and revaccination on outpatient NBPP incidence over 10-year time horizon (% change versus comparator scenario). Impact of vaccine: % change in outcomes for V116 scenarios as compared with equivalent (i.e., matching uptake and revaccination) PCV20 scenarios. Impact of uptake: % change in outcomes for scenarios with 60% uptake as compared with equivalent (i.e., matching vaccine and revaccination) scenarios with 30% uptake. Impact of revaccination: % change in outcomes for scenarios with revaccination as compared with equivalent (i.e., matching vaccine and uptake) scenarios without revaccination.

Scenario number	Impact of vaccine				Impact of uptake				Impact of revaccination			
	overall	18-50	50-60	60+	overall	18-50	50-60	60+	overall	18-50	50-60	60+
Scenario 1												
Scenario 2									-1.7%	-0.7%	-0.7%	-3.9%
Scenario 3					-4.0%	-1.5%	-1.7%	-9.4%				
Scenario 4					-4.9%	-1.8%	-2.1%	-11.8%	-2.6%	-1.0%	-1.1%	-6.5%
Scenario 5	-4.1%	-1.2%	-0.9%	-10.1%								
Scenario 6	-5.3%	-1.2%	-1.0%	-13.4%					-2.9%	-0.7%	-0.8%	-7.5%
Scenario 7	-7.0%	-1.3%	-1.1%	-18.7%	-6.9%	-1.6%	-1.9%	-18.1%				
Scenario 8	-9.0%	-1.4%	-1.2%	-25.3%	-8.6%	-2.0%	-2.3%	-23.9%	-4.7%	-1.1%	-1.2%	-14.1%

Table S21. Comparative impact of vaccine formulation, increased uptake, and revaccination on cumulative outpatient NBPP cases over 10-year time horizon (% change versus comparator scenario). Impact of vaccine: % change in outcomes for V116 scenarios as compared with equivalent (i.e., matching uptake and revaccination) PCV20 scenarios. Impact of uptake: % change in outcomes for scenarios with 60% uptake as compared with equivalent (i.e., matching vaccine and revaccination) scenarios with 30%

uptake. Impact of revaccination: % change in outcomes for scenarios with revaccination as compared with equivalent (i.e., matching vaccine and uptake) scenarios without revaccination.

Scenario number	Impact of vaccine				Impact of uptake				Impact of revaccination			
	overall	18-50	50-60	60+	overall	18-50	50-60	60+	overall	18-50	50-60	60+
Scenario 1												
Scenario 2									-1.8%	-0.7%	-0.8%	-4.3%
Scenario 3					-2.3%	-0.9%	-1.0%	-5.5%				
Scenario 4					-2.7%	-1.0%	-1.1%	-6.5%	-2.2%	-0.8%	-0.9%	-5.3%
Scenario 5	-3.5%	-0.9%	-0.7%	-8.9%								
Scenario 6	-4.7%	-0.9%	-0.7%	-12.4%					-3.1%	-0.7%	-0.8%	-8.1%
Scenario 7	-5.1%	-0.9%	-0.7%	-13.5%	-3.9%	-0.9%	-1.0%	-10.3%				
Scenario 8	-6.7%	-1.0%	-0.8%	-18.4%	-4.6%	-1.0%	-1.2%	-12.9%	-3.8%	-0.9%	-1.0%	-10.7%

Table S22. Distribution of cumulative outpatient NBPP cases covered by PCV20 but not V116, and vice-versa (total cases).

Scenario number	Cumulative IPD PCV20 / non-V116			Cumulative IPD V116 / non-PCV20		
	18-50	50-60	60+	18-50	50-60	60+
	10y	10y	10y	10y	10y	10y
Scenario 1	35,680	12,870	29,699	112,376	53,368	270,671
Scenario 2	35,284	12,625	26,384	112,478	53,429	271,431
Scenario 3	35,202	12,574	25,580	112,498	53,441	271,600
Scenario 4	34,775	12,311	21,895	112,612	53,510	272,444
Scenario 5	38,423	14,018	41,008	107,045	51,358	209,996
Scenario 6	38,436	14,025	41,053	106,698	51,108	190,590
Scenario 7	38,439	14,026	41,064	106,603	51,039	185,053
Scenario 8	38,454	14,034	41,116	106,184	50,737	161,740

References

1. Hethcote HW. The mathematics of infectious diseases. *SIAM review*. 2000;42(4):599-653.

2. Moore MR, Link-Gelles R, Schaffner W, et al. Effectiveness of 13-valent pneumococcal conjugate vaccine for prevention of invasive pneumococcal disease in children in the USA: a matched case-control study. *Lancet Respir Med*. May 2016;4(5):399-406. doi:10.1016/S2213-2600(16)00052-7

3. Whitney CG, Pilishvili T, Farley MM, et al. Effectiveness of seven-valent pneumococcal conjugate vaccine against invasive pneumococcal disease: a matched case-control study. *Lancet*. Oct 28 2006;368(9546):1495-502. doi:10.1016/S0140-6736(06)69637-2

4. Savulescu C, Krizova P, Valentiner-Branth P, et al. Effectiveness of 10 and 13-valent pneumococcal conjugate vaccines against invasive pneumococcal disease in European children: SpIDnet observational multicentre study. *Vaccine*. 2022;40(29):3963-3974.

5. Ryman J, Weaver J, Yee KL, Sachs JR. Predicting effectiveness of the V114 vaccine against invasive pneumococcal disease in children. *Expert review of vaccines*. 2022;21(10):1515-1521.

6. Andrews NJ, Waight PA, George RC, Slack MP, Miller E. Impact and effectiveness of 23-valent pneumococcal polysaccharide vaccine against invasive pneumococcal disease in the elderly in England and Wales. *Vaccine*. 2012;30(48):6802-6808.

7. Suzuki M, Dhoubhadel BG, Ishifuji T, et al. Serotype-specific effectiveness of 23-valent pneumococcal polysaccharide vaccine against pneumococcal pneumonia in adults aged 65 years or older: a multicentre, prospective, test-negative design study. *The Lancet infectious diseases*. 2017;17(3):313-321.

8. Bonten MJ, Huijts SM, Bolkenbaas M, et al. Polysaccharide conjugate vaccine against pneumococcal pneumonia in adults. *N Engl J Med*. Mar 19 2015;372(12):1114-25. doi:10.1056/NEJMoa1408544

9. Lewnard JA, Givon-Lavi N, Dagan R. Effectiveness of pneumococcal conjugate vaccines against community-acquired alveolar pneumonia attributable to vaccine-serotype Streptococcus pneumoniae among children. *Clinical Infectious Diseases*. 2021;73(7):e1423-e1433.
